# Electrochemical Comparison of 2D-Flexible Solid-State Supercapacitors Based on a Matrix of PVA/H_3_PO_4_

**DOI:** 10.3390/polym15204036

**Published:** 2023-10-10

**Authors:** Bianca K. Muñoz, Andrés González-Banciella, Daniel Ureña, María Sánchez, Alejandro Ureña

**Affiliations:** Material Science and Engineering Area, Universidad Rey Juan Carlos, ESCET, C/Tulipán s/n. Móstoles, 28933 Madrid, Spain; andres.banciella@urjc.es (A.G.-B.); duremed.ingenieroenergia@gmail.com (D.U.); alejandro.urena@urjc.es (A.U.)

**Keywords:** flexible supercapacitors, woven carbon fiber, polyvinyl alcohol-based electrolyte, energy storage

## Abstract

Different modifications of woven carbon fiber (WCF) based on carbon aerogel (CAG), copper oxide nanoparticles (CuO-NPs), and lignin (LIG) has been tested and used to study their effect on the fabrication and performance of a flexible supercapacitor. New symmetric flexible supercapacitors (SFSCs) were fabricated using different separators. According to the electrochemical results, the device fabricated using CAG and woven glass fiber (WGF) in a sandwich type configuration CAG/WGF/CAG embedded in H_3_PO_4_/PVA exhibited the best performance (1.4 F/g, 0.961 W/kg, 0.161 Wh/kg). A proof of concept based on a LED powered on and a bending test was done, and the capacitor demonstrated excellent electrochemical values even during and after bending. The new device was able to recover 96.12% of its capacitance when returned to its original unbent position. The manufacturing process was critical, as the fibers or layers must be completely embedded in the gel electrolyte to function effectively. A double flexible supercapacitor connected in parallel was fabricated and it showed higher stability, in the same voltage window, yielding 311 mF/cm^2^ of areal capacitance.

## 1. Introduction

In the last decade, the number of wearable electronic devices including multiple applications, such as watches, suitcases, sports gadgets, clothes, etc., has increased [1,2]. The increasing demand for them in the market has encouraged researchers to study the best method of energy storage by designing flexible batteries (FBs) and supercapacitors (FSCs) that can be modulated, bended, stretched, twisted, or folded without losing their electrochemical performance [3]. It is well-known that supercapacitors are considered a good alternative for energy storage due to the high-power density delivered, which refers to how quickly a device can discharge its energy and is a property usually needed for mobility applications. Fast charge/discharge cycles and relatively easy assembly also make them more attractive [4].

Different strategies to design FSCs have appeared, such as 1D (coaxial fibers) [5], 2D (thin films or sandwich type) [6], and 3D (hierarchical frameworks) [7] and they have been applied to different materials such as carbonous fibers [8], textile fibers (polyester) [9], flexible papers and foams [10,11], and woven fibers [12]. Carbon cloth (CC) is a very convenient textile due to its high conductivity performance, price, flexibility, and feasibility of surface modification to enhance its behavior as electrodes [13]. Electrical double layer capacitors (EDLCs) are highly dependent on the surface-interface character of the electrodes. For this reason, carbon fiber surface modifications by oxidation treatments [14] or by the incorporation of carbon nanostructures such as carbon nanotubes (CNTs) [11] or graphene nanoplatelets (GNPs) [15] have previously been reported. Moreover, the modification by 3D metal oxide arrays on the carbon cloth has also been reported as well as combinations of carbon nanostructures and metal oxides [14]. Other interesting modifications include some wastes from biomass as lignocellulosic biomass-derived carbon [16], which are used directly as electrodes due to the high porosity and hierarchical structures. In other cases, the use of lignin-modified carbon fiber was reported to improve the interfacial adhesion in carbon fiber-reinforced polymers [17]. 

By exfoliation and oxidation of the surface of CC (modified Hummer’s method) using KMnO_4_, H_2_SO_4_, and HNO_3_ solution, a symmetric FSC fabricated with sulfuric acid and poly(vinyl alcohol) (PVA) gel electrolyte showed a capacitance of 31 mFcm^−2^ (0.0015 Fg^−1^) at a scan speed of 10 mVs^−1^ [18]. Liu and Zhou included a carbon nanotube 3D network on CC for a FSC assembled using KOH/PVA, obtaining a capacitance of 106.1 Fg^−1^, an areal capacitance of 38.75 mFcm^−2^, and an ultralong cycle life of 100,000 times (capacitance retention: 99%) [19]. Symmetric FSCs using carbon fabric modified with metal oxides as electrodes have also been efficiently developed. MnO_2_ has been extensively used to modify the surface in symmetric FSCs, an areal capacitance of 42.4 mF/cm^−2^ at 5 mVs^−1^ has been reported. Moreover, a FSC using manganese dioxide nanorod arrays on carbon fiber was fabricated using PVA/H_3_PO_4_ as electrolyte [20]. The modification of the fiber showed a very good performance (678 Fg^−1^ at a current density of 0.3 Ag^−1^). For heterostructure nanoarrays with CuCo_2_O_4_@MnO_2_ nanowire-based electrodes, a high cell-area-specific capacitance of 714 mFcm^−2^ at 1 mAcm^−2^ was achieved for the device using (PVA)/KOH polymer electrolyte [21]. The Co_3_O_4_ nanoparticles on vertically aligned graphene nanosheets (VAGNs) were also tested for FSC (PVA/KOH as electrolyte), delivering high capacitance 580 Fg^−1^ and high energy density (80 Wh/kg) and power density (20 kW/kg) [14]. CoMoO_4_@NiCo-layered double hydroxide nanowire arrays have also been used to modify CC and led to the fabrication of asymmetric FSC with PVA/KOH as electrolyte, exhibiting a maximum energy density of 59 Whkg^−1^ at a power density of 800 Wkg^−1^ [22]. CuO-based nanomaterials have been found to be very attractive for supercapacitor applications due to their low cost, facile and reproducible preparation methods, and electrochemical responses [23]. It is well known that light-weight carbon aerogel (CAG) has some characteristics that make them ideal for SC electrodes, such as large surface area, low mass density, and high porosity [24,25]. 

The separator also plays an important role in the supercapacitor performance and flexibility. In the flexible supercapacitors approach, some authors suppress the use of them because the polymer electrolyte can act as a dielectric. In other cases, authors prefer to use separators based on woven glass fiber, woven glass fiber (WGF), filter paper mat (FP), and, for structural applications, Kevlar^®^ fiber fabric [26].

As it has been previously mentioned above, one of the most interesting approaches for the development of flexible supercapacitors is the use of woven carbon fiber as electrodes. The carbon cloth has some characteristics that make it attractive to be used as an electrode from an economic and environmental point of view. An appropriate combination of electrodes based on carbon fabric with PVA/H_3_PO_4_ matrix electrolyte can make the development of new supercapacitors for smart applications possible, being scalable and supporting their increasing demand. In general, the electrodes developed so far showed outstanding properties. However, the modified electrodes have complicated fabrication processes and usually they are very expensive, not to mention that in some cases, they are still far from being used for immediate applications. In this sense, modifying carbon fabric in an easy and cheap manner represents a more challenging goal. Moreover, despite the great number of studies in FSCs using CC as substrate electrode, their performance is not easy to compare and unify due to different ways of fabrication (assembling, separator, and electrolyte) and characterization.

In this work, we present the modification and characterization of woven carbon fiber (WCF) including carbon aerogel (CAG), copper oxide nanoparticles (CuO NPs), and lignin (LIG), as well as their application as electrodes for flexible supercapacitors using PVA/H_3_PO_4_ as gel polymer electrolyte. We tested their performance before, during, and after bending tests to demonstrate their flexibility and ability to recover the electrochemical response. The electrochemical performance of the devices is discussed, and a proof of concept based on a red LED powered on was done with the system WCF/WGF/WCF.

## 2. Materials and Methods

### 2.1. Materials

Woven carbon fabric HexForce^®^ 48,193 plain 12 K, manufactured by Hexcel (Stamford, CT, USA), was used in this work. The separator used was of filter paper (FP) or woven glass fiber (WGF). The glass fiber separator (E-Fiberglass Woven Roving) was supplied by Castro Composites^®^ (Pontevedra, Spain). Glass Filter paper Whatman, Lignin alkaline (low sulfonate content), resorcinol, formaldehyde (37 wt% in H_2_O), copper acetate dihydrate, copper chloride, (L)-ascorbic acid (L-AA), PVP K90, K_2_CO_3_, KOH, H_3_PO_4_ (≥85 wt% in H_2_O), and Poly(vinyl alcohol) (PVA, Mw 89,000–98,000, 99+% hydrolyzed) were purchased from Sigma-Aldrich (St. Louis, MO, USA). Liquid sizing agent (SICIZYL) was purchased from Nanocyl S.A. (Sambreville, Belgium). The CuO seed on carbon cloth was done following the procedure previously reported in the literature [27]. The carbon aerogel modification was prepared following the procedure reported [28].

### 2.2. Preparation and Characterization of Electrodes

In all cases, fabric was washed with ethanol before any particle deposition and dried in the oven at 60 °C. 

#### 2.2.1. Dip-Coating (DC) of Lignin

A water suspension of lignin (10 wt%) in distilled water and sizing solution (5 wt%) was stirred at room temperature for 30 min. Then, the carbon fiber fabric was soaked for 10 min and dried in the oven at 80 °C for 10 more minutes until solvent evaporation. This procedure was repeated twice.

#### 2.2.2. Hydrothermal Growth of CuO Nanoparticles

The hydrothermal method used here is a modification of a procedure previously reported [29], using an optimized mixture of CuCl_2_/NaCl/L-AA of 1:3.5:15 in DIW (375 mL) containing 1 wt.% of PVP K90. A solution of CuCl_2_ was stirred in 150 of DIW for 20 min. In a different vessel, L-AA was also dissolved in 200 mL of DIW under sonication for 30 min, then mixed with NaCl and PVP-K90 and stirred. The copper chloride solution was added to AA solution under stirring and the pH was adjusted using NaOH. The seeded WCFs were immersed in the growth solution into the stainless-steel autoclave in an oven at 80 °C. The desired growth temperature for the CuO/WCF hybrid at a fixed growth time of 2 h. After the hydrothermal processing, the CuO/WCF hybrid was rinsed with DIW to stop further growth of CuO NPs and dried at room temperature for 1 day. 

#### 2.2.3. Surface Morphology and Area Characterization

The specific surface area of the modified carbon fiber fabric was calculated by the Brunauer, Emmett, and Teller (BET) method based on N_2_ adsorption–desorption isotherms recorded with a Micrometrics ASAP 2020 analyzer. Fabric surface characterization was also performed evaluating the images from Scanning Electron Microscopy (SEM, S-3400 N from Hitachi, Tokyo, Japan) and Field Emission Gun SEM (FEG-SEM, Nova NanoSEM FEI 230 from Philips, Amsterdam, The Netherlands). Samples were previously coated by a 6 nm layer of gold for proper characterization.

### 2.3. Electrochemical Characterization of Electrodes

Cyclic voltammetry (CV) tests on modified carbon fibers were performed at room temperature with a three-electrode cell, using an Ag/AgCl reference electrode and a platinum counter electrode in 3 M KCl aqueous electrolyte. The working electrode consisted of a single tow partly immersed into the electrolyte and electrically contacted at the dry end. Tows were measured and weighed before the test and the active mass (*ma*) of the working electrode was determined from the immersed length.

Experiments were conducted using an Autolab PGSTAT302N system. Different scan rates (from 10 to 100 mV/s) were tested on a representative specimen from each condition. The specific capacitance (*C_sp_*) was calculated with Equation (1).

(1) $$Csp=\frac{\int_{v}^{vf}IdV}{s\cdot ∆V\cdot ma}$$

where *I* is the current (A), *dV* is the differential voltage corresponding to both charge and discharge processes between final voltage (*vf*) and initial voltage (*v*), *ma* (g) is the mass of the electrodes, *s* (V s^−1^) is the scan rate, and ∆*V* is the potential window of the *CV*. Representative capacitance results were calculated using a potential window (Δ*V*) from −0.1 to 0.1 V and a scan rate (*s*) of 10 mV/s.

### 2.4. FSC Fabrication

The gel polymer electrolyte (GPE) was prepared by mixing H_3_PO_4_ (3 g) and PVA (3 g) in distilled water (30 mL) [30] under stirring at 85 °C for 30 min or until the solution became clear. The electrodes (previously modified and attached to a copper current collector wire glued using silver ink) and separator (glass fiber or filter paper) were immersed in a bath containing the GPE mixture for a few minutes to favor the impregnation. Then, the device was sandwich-type assembled following the configuration electrode/separator/electrode over a metallic mold and left to cast a 60 °C in the oven for 8 h.

### 2.5. Electrochemical Characterization of FSCs

The electrochemical characterization of the symmetric SC device was measured in a two-electrode setup. Cyclic voltammetry (CV) tests on FSCs were carried out by using a potentiostat AUTOLAB PGSTAT302N with a software Nova 2.1. Different scan rates (from 10 to 100 mV/s) were tested on a representative specimen from each condition. Each sample was subjected to 5 consecutive voltammetry cycles to determine its specific capacitance using Equation (1). Representative capacitance results were calculated using a potential window (Δ*V*) from −0.5 V to 0.5 V and a scan rate (s) of 10 mV/s. Galvanostatic charge-discharge tests (GCD) and electrochemical impedance spectroscopy (EIS) tests were carried out to characterize in depth their capacitor capabilities. From GCD analysis, specific capacitance (*C*, F/g) can be derived from Equation (2), while energy density (*E*) and power density can be obtained according to Equations (3) and (4) [22]. EIS was performed in samples in a frequency range of 10^5^–0.1 Hz. Both tests were also performed in an AUTOLAB PGSTAT302N module with a software Nova 2.1.

(2) $$C=\frac{I\times ∆t}{\begin{array}{c} m\times ∆V \end{array}}$$

(3) $$E=\frac{1}{2}C\times {∆V}^{2}$$

(4) $$P=\frac{E}{∆t}=\frac{1}{2}\frac{I\times ∆V}{m}$$

where *I* is the current (A), ∆*t* (s) the discharge time, *m* (g) the electrode mass, and ∆*V* the potential window.

## 3. Results and Discussion

### 3.1. Electrodes Modification and Characterization

**Figure 1:**
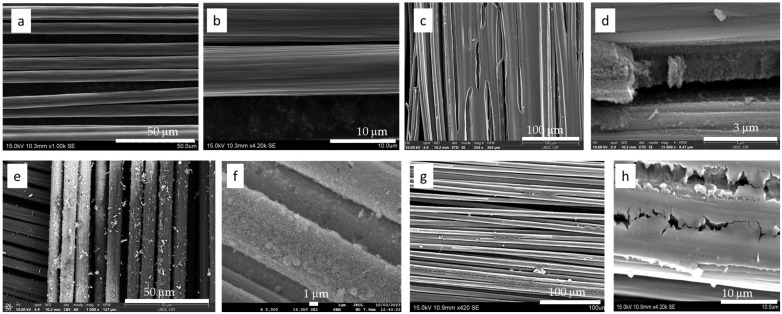
SEM images of modified carbon fibers. (**a**,**b**) Two magnifications of bare carbon fiber, (**c**,**d**) two magnifications of carbon fiber modified with carbon aerogel, (**e**,**f**) two magnifications of carbon fiber modified with CuP NPs, (**g**,**h**) two magnifications of carbon fiber modified with lignin.

The surface morphology of the new modified carbon fibers was investigated by FEGSEM (Figure 1). 

In this image, the plain WCF appears as a corrugated non-porous surface. The images of modified fibers containing CAG, CuO, and lignin are shown in Figure 1c–h. For the sample modified with CAG (Figure 1c,d), the covering looks like a thick layer; however, in the closer picture, it is possible to observe a porous surface in this layer. The modification with CAG is done from carbonization RF polymer directly cured over the CC; then, the CAG layer thickness depends on the pressing step during the cured. Even though the CAG layer is highly porous as can be demonstrated from the specific surface area (SSA) of the electrode reported in Table 1 (BET area), the CuO NPs growth over the carbon fiber shows a more homogeneous layer (Figure 1e,f). The SSA is not as high as those values obtained for CAG but is higher than the pristine WCF (0.244 m^2^ g^−1^). The SEM images of fiber modified using lignin by dip-coating (Figure 1g,h) show a brittle organic layer corresponding to lignin and sizing (commercial organic sizing). The SSA obtained for this sample is even smaller than the pristine WCF, meaning the lignin by itself is not porous, and is not conferring any extra surface area.

The electrodes were analyzed by cyclic voltammetry at different scan rates and the specific capacitance was determined at 10 mVs^−1^. The specific capacitances of the electrodes are shown in Table 1.

**Scheme 1:**
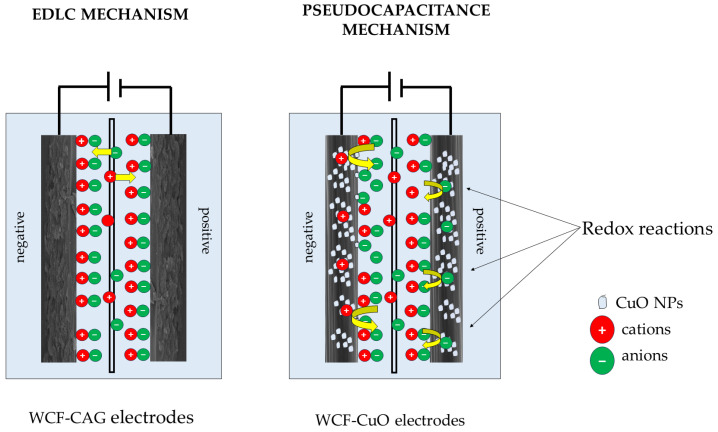
Schematic illustration of EDLC (**left**) and pseudocapacitance (**right**) mechanisms.

As could be expected, the specific capacitance for the pristine WCF is low (0.19 Fg^−1^). As the BET surface area increased for the electrodes modified with CuO-NPs or CAG (entries 2 and 3), it is evident that the *C_sp_* increased, reaching values between 1.5 and 5 Fg^−1^. The CAG on WCF was previously studied by different authors using EMIMTFSI as an electrolyte and the specific capacitance reported is in the same order of magnitude (2.4 F/g) [31]. In the case of WCF-CuO, it has been previously reported that depending on the structure of the NPs, they can provide either large BET area and behave as an EDLC [32] or they can present certain pseudocapacitance as a dominant energy storage mechanism. In the last case, the electrodes can record high capacitances through a Faradaic process. For transition metal oxide nanostructures, the effect of the morphology on the electrochemical behavior is quite remarkable [23]. A schematic illustration of both EDLC and pesudocapacitor charge storage mechanism are depicted in Scheme 1. Pseudocapacitors usually exhibit better capacitance than double layer capacitors [33].

The sample modified with lignin showed a very low specific capacitance, even lower than the pristine WCF. This value can be attributed to the isolating nature of the sizing used as a binder, which is an organic oligomer. This effect when polymeric binders or surfactants are used has been previously reported [34].

With these electrodes, symmetric flexible supercapacitors were fabricated using PVA/H_3_PO_4_ as gel polymer electrolyte and using a separator of filter paper (FP).

### 3.2. Flexible Supercapacitors’ Electrochemical Characterization

**Figure 2:**
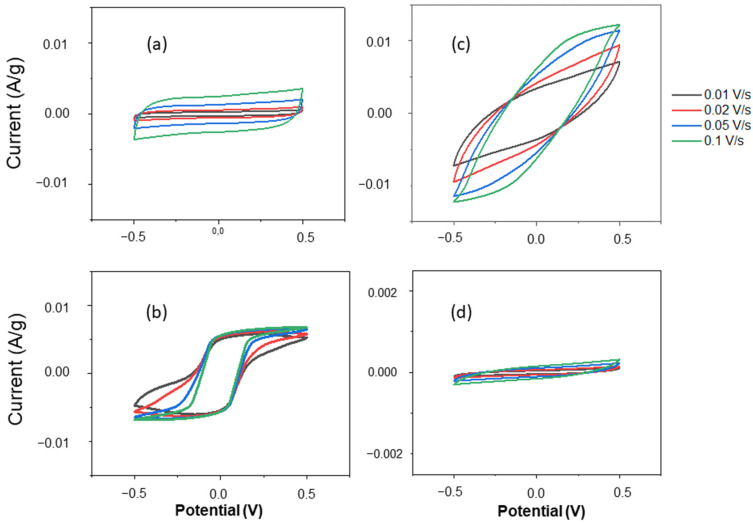
CV of the FSCs fabricated from the modified electrodes. (**a**) Bare carbon fiber electrodes, (**b**) CuO NPs modified electrodes, (**c**) CAG modified electrodes, (**d**) lignin modified electrodes.

The modified carbon fiber cloths were used as electrodes to fabricate the flexible supercapacitors. The electrolyte was prepared, and the electrodes and separator were dipped in for a few minutes. Then, they were piled up using a sandwich type configuration by placing the layers in the following order: electrode/separator/electrode. The FSCs obtained were initially characterized by CV and EIS (Figure 2 and Figure S1).

The CV of FSC with the bare WCF and those modified with CuO NPs or CAG have the same scale, while the CV of FSC fabricated using the electrodes modified with lignin is shown in a different scale. The CV curves show how the surface modifications have affected the electrochemical performance of the devices. The FSC fabricated using plain WCF exhibits good electrochemical stability at different potentials. In this case, the area inside the CV curve is small but the shape indicates typical capacitive non-Faradaic behavior.

The specific capacitances were normalized by the electrode masses due to the different additional weights corresponding to the electrolyte (as they do not contain the same amount and not all the gel electrolyte is in contact with the electrodes). The FSCs modified either with CAG or CuO NPs show higher specific capacitance since the area inside the curve is higher. The electrochemical values obtained from these curves are shown in Table 2.

Both FSCs, either with CuO NPs or CAG, present similar specific capacitances (from 0.26 to 0.33 F/g at 10 mV/s). The areal capacitances are comparable to systems modified with Mn oxides using KOH in PVA as electrolytes [35]. Regarding the electrochemical stability, the FSC using electrodes modified with CuO seems to be less stable in the same potential window compared to CAG, which shows less prominent vertical changes. It has been reported that some zero-dimensional nanoparticles of pristine copper oxide could show poor electrochemical stability and high tendency towards rapid capacity decay [23]. In Figure 2b, at the lower scan rate (0.01 Vs^−1^), it is possible to detect some deviations in the CV, suggesting some Faradaic processes; these small humps disappear at faster scan rates. However, more studies are currently in progress to obtain more information about the plausible energy storage mechanism followed by these CuO nanoparticles. In the case of FSC modified with WCF-LIG, the specific capacitance of the devices are two orders of magnitude smaller than those obtained for CuO and CAG electrodes, consistent with the results obtained for plain electrodes analyzed in KCl. Concerning the results extracted from the EIS characterization (Figure S1), the EIS curves show a depressed semicircle at high frequencies associated with the ion transport processes, followed by a spike at low frequencies. The semicircle for the FSC-CuO is smaller, meaning that there is less resistance due to the best surface interface contact between the electrode and the matrix as it has been reported for similar CuO-WCF electrodes using structural epoxy matrices. The FSC-CAG shows a larger spike that is translated into a more capacitive behavior, typical for porous carbon electrodes. As a counterpart, the FSC-LIG exhibits high resistance values. The *R_s_* corresponds to the ionic transport through the electrolyte and electrode. That means that the values of *R_s_* can be directly related to the ionic conductivity capabilities. The isolating sizing layer present in the LIG electrodes led to an increase in the resistance values, diminishing the ionic movement between the electrolyte/electrode and affording an extremely low specific capacitance value. The conductivity of the electrolyte in the flexible device modified with CuO is slightly higher than the obtained for the electrode modified with CAG, due to a better interface interaction that exhibits less resistance to the ion transport. The value corresponding to the FSC fabricated with the bare WCF is shown in Table 2 for comparison.

### 3.3. Bending Tests

**Figure 3:**
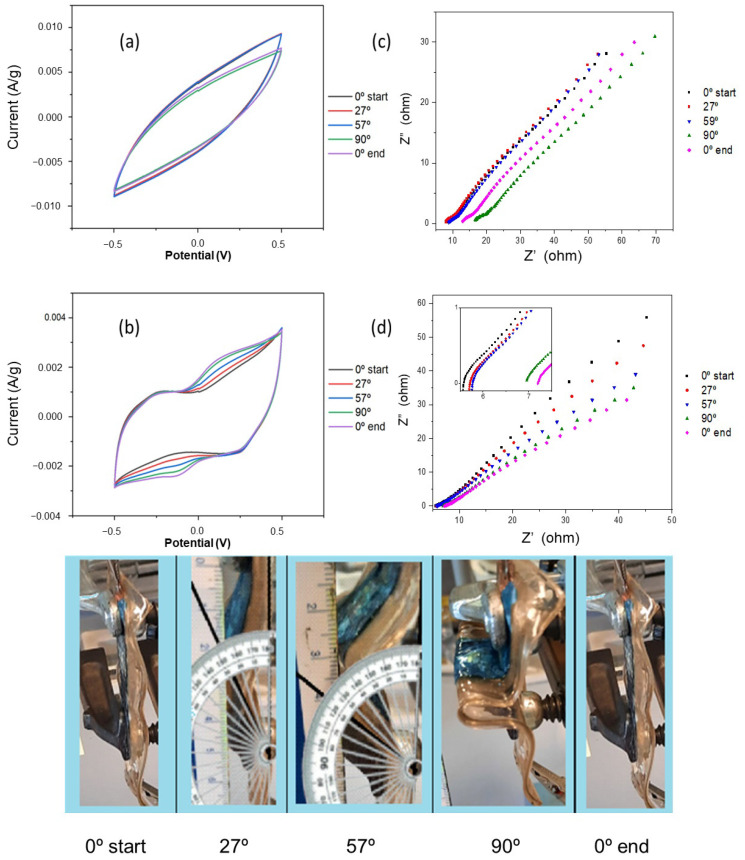
CV and EIS analysis of the FSCs during the bending test. (**a**,**c**) for CAG/CC and (**b**,**d**) for CuO/CC. The picture shown of the bending angle corresponds to the sample modified with CAG.

To demonstrate their application as flexibles devices, their flexibility was evaluated by bending and analyzing the FSC by CV. The results are shown in Figure 3. 

The absence of significant distortions in the shape of the CV curves at the different bending angles for both FSCs reveal high flexibility and certain stability of both devices. The resistances are more affected instead, revealing a higher resistance after the bending. According to the internal area of the CV curves, it was possible to calculate the capacitance retention (%) during and after bending (Figure S2). For FSC-CuO and FSC-CAG, the behavior was similar. At 27°, both devices retained >95% of the capacitance; at 57°, CAG-FSC retained 96% and CuO-FSC 93%; and at 90°, the capacitance dropped to 80% in both devices, but they recovered their capacitance over 85% when returned to the initial position. The picture at the bottom of Figure 3 shows the dramatic bending at 90°. For the sample FSC-LIG, the capacitance retention fell to 69% at 90° and in general, it exhibited a worst performance.

**Figure 4:**
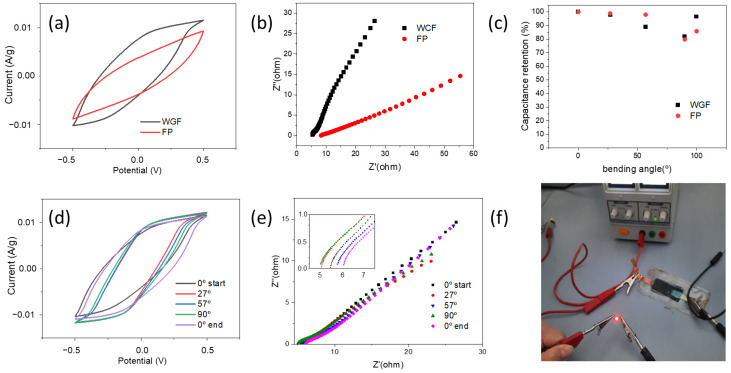
Characterization of the new device (CAG/separator/CAG) using a different separator (WGF). (**a**–**c**) Electrochemical performance comparison of FSCs. (**d**,**e**) CV and EIS of the device fabricated using woven glass fiber separator. (**f**) Proof of concept of red LED switched on.

A new device was fabricated using a different separator, woven glass fiber (WGF), to compare its performance with the filter paper (FP). The CV at 10 mVs^−1^ and EIS analysis shown in Figure 4 were used to characterize the device before, during, and after bending. The FSC-CAG fabricated using WGF as separator showed better electrochemical values, such as less resistance and a higher specific capacitance. The capacitance retention (%) at different bending angles for the flexible supercapacitors fabricated with WGF or FP as separator indicated that there were not big differences in using both separators. However, after the bending, the device using WGF recovered 96% of the initial capacitance, showing that it is more flexible. The GCD tests were also measured for both samples (Figures S3 and S4) and the ratio for the device modified using the FP separator had a t_discharge_/t_charge_ ratio of 0.62 compared to the 0.47 value obtain for the sample separated using WCF. The specific capacitance and other electrochemical parameters calculated from EIS and GCD tests (at 2 mA g^−1^) are listed in Table 3.

### 3.4. Proof of Concept

**Figure 5:**
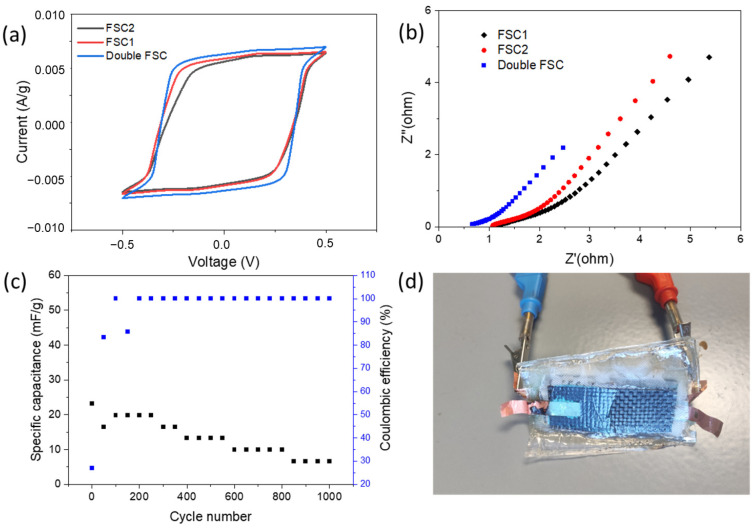
Electrochemical characterization of the double FSC-CAG connected in parallel, and each device separated (FSC1 and FSC2). (**a**) CV curves; (**b**) EIS analysis; (**c**) CDG results; and (**d**) double FSC device.

The modified device using CAG electrodes and WGF separator was charged for 30 min at 2 V and was able to switch on a 1.6 V red LED, but only for a few minutes (1 min of bright light) (Figure 4f). This short time agrees with the fast discharge previously observed during the CDG. Despite the short time of the proof, this device kept 83.34% capacitance at 90° of bending and recovered 96.12% when returned to the initial unbending position. A new device was prepared following the same procedure but coupling two devices connected in parallel. For this fabrication, the modified CAG electrodes corresponded to the same batch to avoid the introduction of new variables. The CV and EIS curves obtained from the electrochemical characterization of the double and singles devices are depicted in Figure 5.

As can be observed, the square shape of the three CV curves is consistent with an ideal non-Faradaic capacitive behavior, and it shows higher stability in the voltage window analyzed. The results obtained from the CDG and EIS test are listed in Table 4.

The areal capacitance for this new device was 0.311 F/cm^2^, much higher than the results obtained for the FSC-CuO, and the resistances observed from the EIS analysis (*R_s_* and *R_ct_*) were exceptionally low compared to the single devices previously fabricated. The better electrochemical values obtained for the double FSC-CAG can be attributed to a better impregnation of the electrodes with the gel electrolyte during the fabrication, which led to the electrodes being better embedded in the matrix. This better encapsulation could favor the ion migration and transport, increasing the ionic conductivity and specific capacitance.

Two important aspects about the fabrication method must be considered: (1) The fabrication method of these devices can be a limitation, because depending on how good it is, there may be a different performance from one batch to another. (2) There are too many factors affecting the fabrication such as covering layer thickness, surface area and porosity, cured process, electrolyte impregnation, and casting process, which must be controlled to improve reproducibility and stability.

Even though these results are far from being scaled up, they demonstrate that the FSCs fabricated in this study exhibit better performance and capacitance than other FSCs made from carbon aerogel on woven carbon fiber. Of various sources of carbon that can be used to create potential electrodes for flexible applications, carbon fabric remains an ideal substrate despite its low specific surface area. The carbon aerogel synthesized from RF resin produces a highly porous covering that can modify the carbon cloth in a less expensive manner compared to other different carbon nanostructure approaches. Recent studies have focused on the use of CAG on CF to study their performance in structural supercapacitors [36].

Some results obtained for CAG/WCF electrodes using different electrolytes are depicted in Table 5 for comparison with the results obtained in this study. These results are also compared with those for bare carbon fiber, activated carbon fiber (etched and grafted), and other relevant carbonaceous coverings such as CNTs or GNP.

The FSCs prepared in this study are competitive, highly flexible, and low cost, which is an important remark. It is expected that the materials developed in this work will have practical value and interest. Further studies involving more efficient fabrication methods are currently underway.

## 4. Conclusions

This study explores the effect of modifications made to woven carbon fiber on the performance of flexible supercapacitors. The electrodes were modified by the incorporation of copper oxide nanoparticles (CuO-NPs), carbon aerogel (CAG), and lignin covering (LIG) as coverings. Reinforced carbon fiber electrodes were employed to fabricate 2D-flexible supercapacitors using a sandwich configuration of CF-electrode/separator/CF-electrode, all coated with H_3_PO_4_/PVA gel electrolyte. The FSC produced a specific capacitance of 1.4 Fg^−1^, which is comparable to other supercapacitors outlined in the existing literature. This translated into energy and power densities of 0.161 Wh kg^−1^ and 0.961 W kg^−1^, respectively. The CAG/WGF/CAG configuration showed a *R_s_* of 5.48 ohm and demonstrated excellent electrochemical values, even during and after bending. The capacitor was able to recover 96.12% of its capacitance when returned to its original unbent position. In this study, the use of woven glass fiber (WGF) as a separator was found to improve the electrochemical performance. Finally, the fabrication process is critical, as the fibers must be embedded thoroughly in the gel electrolyte to perform effectively.

## Figures and Tables

**Table 1:** T BET areas and specific capacitances of carbon fibers.

Entry	Sample	S_BET_ (m^2^/g_C+cover_)	*C_sp_* (F/g_C+cover_)
1	WCF	0.244	0.19
2	CuO	0.853	5.42
3	CAG	63.47	1.7
4	LIG	0.278	0.18

**Table 2:** Electrochemical data from CV and EIS analysis of FSCs.

SCF	*C_sp_* (mF/cm^2^)	*C_sp_* (F/g)	σ (mS cm^−1^)	*R_s_* (Ω)	*R_s_* + *R_ct_* (Ω)
FSC-WCF	-	0.023	-	-	-
FSC-CuO	12.1	0.328	2.08	2.95	10.61
FSC-CAG	9.6	0.259	1.44	8.26	69.10
FSC-LIG	0.14	0.004	0.034	164.9	414.68

**Table 3:** Electrochemical performance of FSCs with different separators.

Separator	*R_s_*/*R_s_* + *R_ct_* (Ω)	*C_sp_* (F/g)	P (W/kg)	E (Wh/kg)
FSC-FP	8.26/69.1	0.563	0.815	0.048
FSC-WGF	5.48/8.48	1.369	0.962	0.161

**Table 4:** Electrochemical data of flexible supercapacitors connected in parallel.

Device	Capacitance (F)	Areal C (F/cm^2^)	*R_s_*/*R_ct_* (W)
FSC1	2.14	0.143	1.02/1.14
FSC2	2.00	0.134	1.13/1.36
Double FSC	4.66	0.311	0.6/1.13

**Table 5:** Electrochemical data from CV analysis of FSCs.

Composites	Electrolyte	Electrodes (F/g)	Capacitor	Reference
WCF	3 M KCl	0.02	-	This work
WCF	3 M KCl	0.06	-	[25]
a-WCF	3 M	2.63	-	[25]
a-CFC	6 M KCl	197 (0.1 A/g)	0.5 Fcm^−2^	[37]
ACF	PVA-H_3_PO_4_	18.6	300 mFcm^−2^	[36]
WCF-CNT	EMIMTFSI	13.26	0.010	[38,39]
WCF-CNT	3 M KCl	0.015		[28]
WCF-GNP	3 M KCl	0.405		[28]
WCF/CAG	EMIMTFSI	2.4	-	[31]
WCF/CAG	EMIMTFSI	11.4	1.74 Fg^−1^; 135 mFcm^−2^	[40]
WCF/CAG	Epoxy resin/EMIMTFSI	-	0.212 Fg^−1^	[40]
WCF/CAG	3 M KCl	5.9	-	[25]
CF/CAG	EMIMTFSI	-	1.49 Fg^−1^	[25]
CF/CAG	EMIMTFSI	-	0.75 Fg^−1^	[41]
WCF/CAG	3 M KCl	4.35	1.4 Fg^−1^	This work
WCF/CuO	3 M KCl	5.42	0.33 Fg^−1^	This work

## Data Availability

The data presented in this study are available on request from the corresponding author.

## Supplementary Materials

The following supporting information can be downloaded at: https://www.mdpi.com/article/10.3390/polym15204036/s1, Figure S1: EIS analyses of the FSCs. CuO (Blue), CAG (Black), and LIG (Green); Figure S2: % Capacitance retention before, during and after bending for the FSCs analyzed; Figure S3: GCD tests for the samples FSC-CAG/FP/CAG (a and c) and FSC-CAG/WGF/CAG (b and d) at 0.002 A g^−1^; Figure S4: GCD tests for the doubled FSC CAG/WGF/CAG/CAG/WCF/CAG at different scan rates.
