# Supplementary material for: Electrochemical Comparison of 2D-Flexible Solid-State Supercapacitors Based on a Matrix of PVA/H3PO4
Source: Polymers (Basel). 2023 Oct 10;15(20):4036. doi: 10.3390/polym15204036 (PMC10610347; doi:10.3390/polym15204036)
Supplement: Supplementary file 1 [file polymers-15-04036-s001.zip › polymers-2640722-supplementary.pdf]

Electrochemical Comparison of 2D-Flexible Solid-State Supercapacitors Based on a Matrix of PVA/H_3PO_4

Bianca K. Muñoz *, Andrés González-Banciella, Daniel Ureña, María Sánchez * and Alejandro Ureña

Figure		Page
**Figure S1**	EIS analyses of the FSCs. CuO (Blue), CAG (Black) and LIG (Green)	2
**Figure S2**	% Capacitance retention before, during and after bending for the FSCs analyzed	2
**Figure S3**	GCD tests for the samples FSC-CAG/FP/CAG (a and c) and FSC-CAG/WGF/CAG (b and d)	3
**Figure S4**	GCD tests for the doubled FSC CAG/WGF/CAG/CAG/WCF/CAG at different scan rates	3

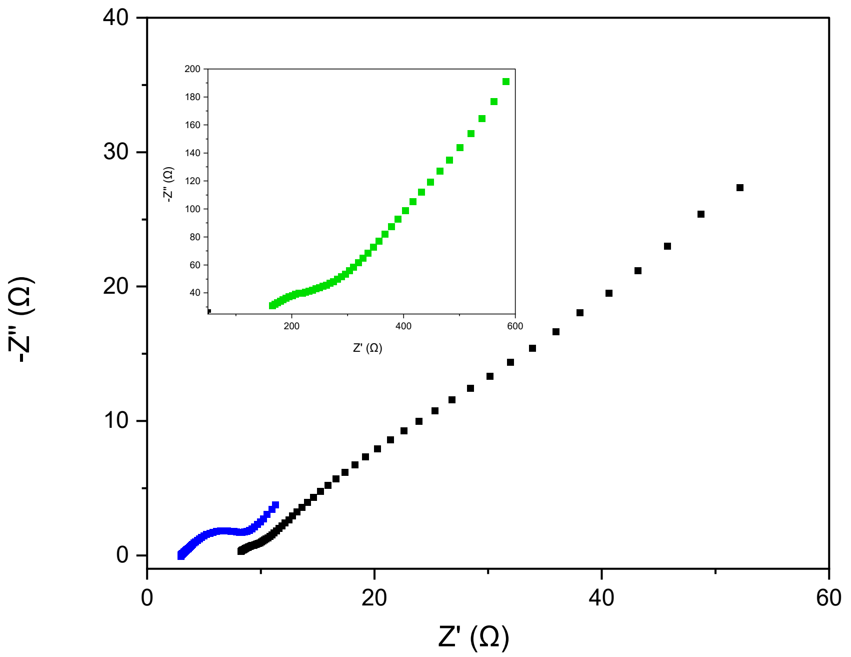

Figure S1. EIS analyses of the FSCs. CuO (Blue), CAG (Black) and LIG (Green)

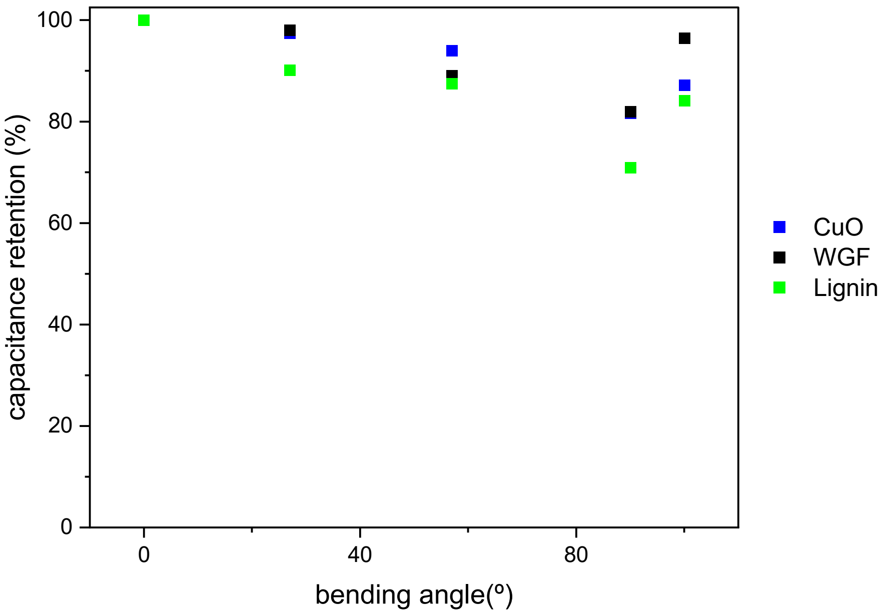

Figure S2. % Capacitance retention before, during and after bending for the FSCs analyzed.

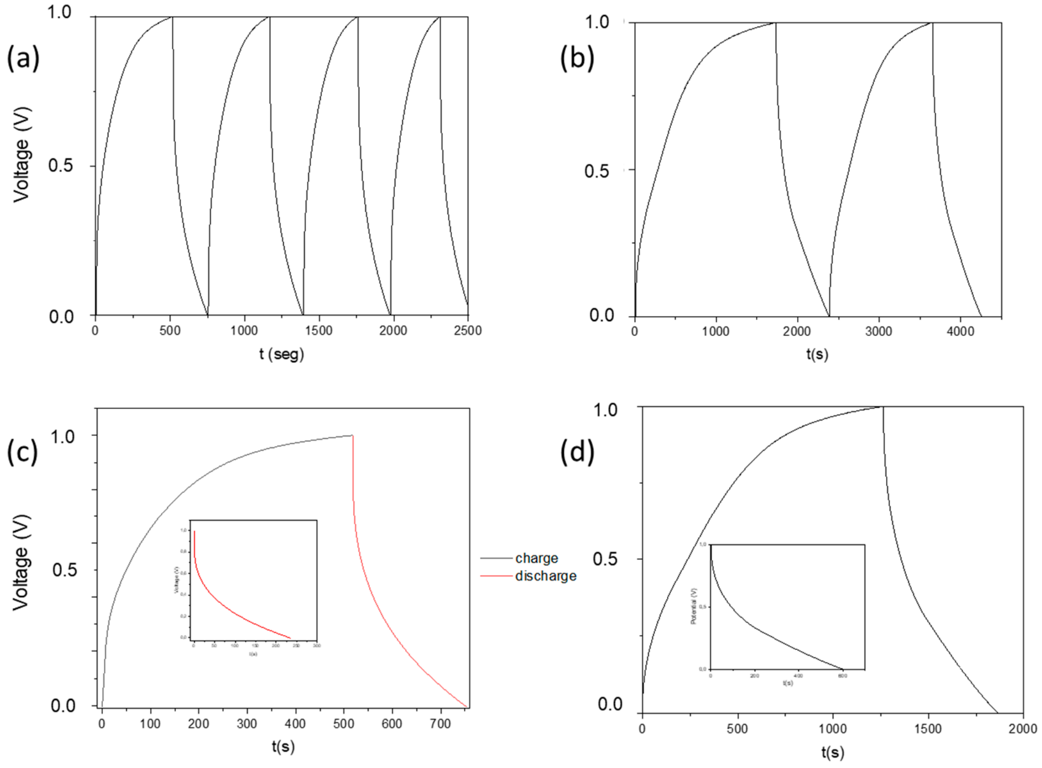

Figure S3. GCD tests for the samples FSC-CAG/FP/CAG (a and c) and FSC-CAG/WGF/CAG (b and d) at 0.002 A g^{-1}

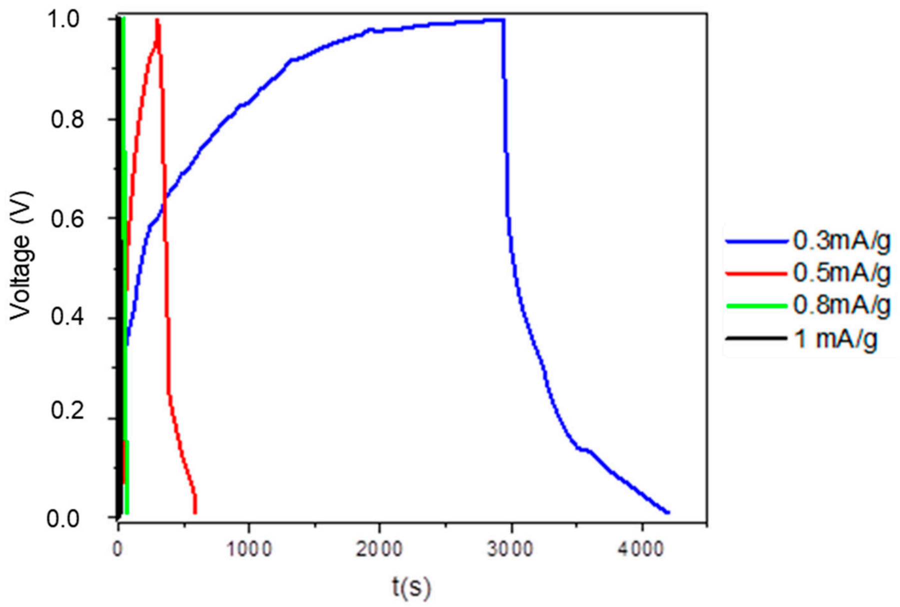

Figure S4. GCD tests for the double FSC-CAG/WGF/CAG at different scan rates
